# Supplementary material for: Comparative Transcriptomics in Atlantic Salmon Head Kidney and SHK-1 Cell Line Exposed to the Sea Louse Cr-Cathepsin
Source: Genes (Basel). 2023 Apr 13;14(4):905. doi: 10.3390/genes14040905 (PMC10138087; doi:10.3390/genes14040905)
Supplement: Supplementary file 1 [file genes-14-00905-s001.zip › Supplementary Figures.pdf]

Supplementary figure list

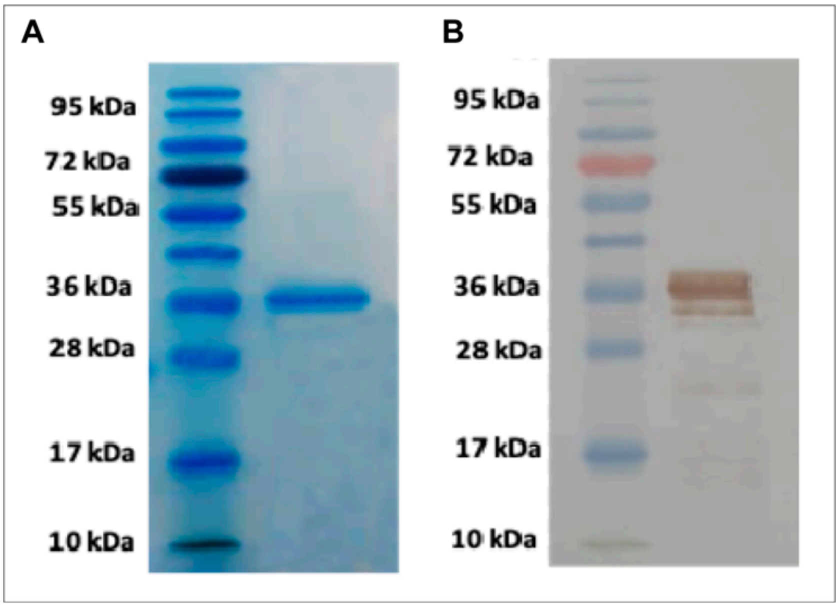

Figure S1. Purification of recombinant Cr-Cathepsin protein by affinity chromatography. (A) SDS-PAGE 12%. (B) Western blotting Mab α-His HRP-conjugated. Lane 1: Molecular weight marker Pre-stained SDS-PAGE Standards Broad Range (BioRad, USA).

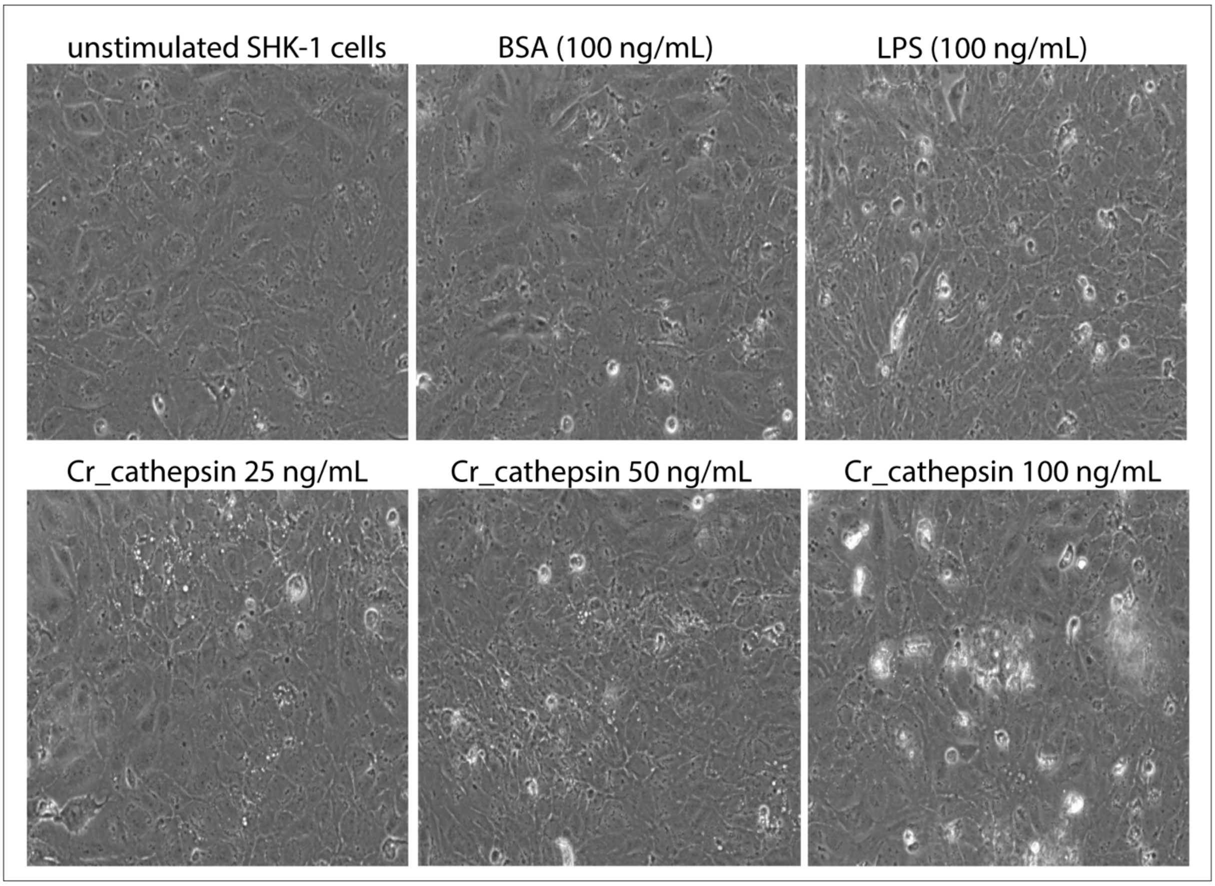

Figure S2. Effect of recombinant Cr-Cathepsin (25, 50, 100 ng/mL) and controls (BSA 100 ng/mL and LPS 100 ng/mL) in SHK-1 cell line after 24 hours of treatment using EVOS^{TM} M5000 Imaging System microscope (Thermo Fisher Scientific, USA). Bright field, 40X magnification.

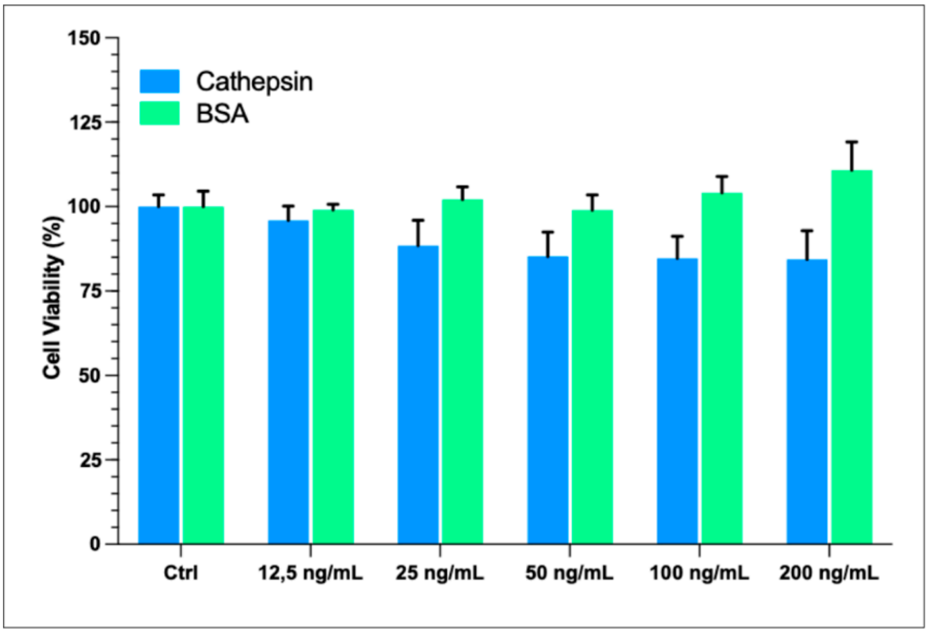

Figure S3. Effect of recombinant Cr-Cathepsin in the viability of SHK-1 cells. Two-fold dilutions of Cr_cathepsin and BSA were used, starting from 200 to 12,5 ng/mL. Viability was measured by alamarBlue^{TM} assay. The viability was plotted as the difference in percent reduction in emitted fluorescence between treated and control cells.

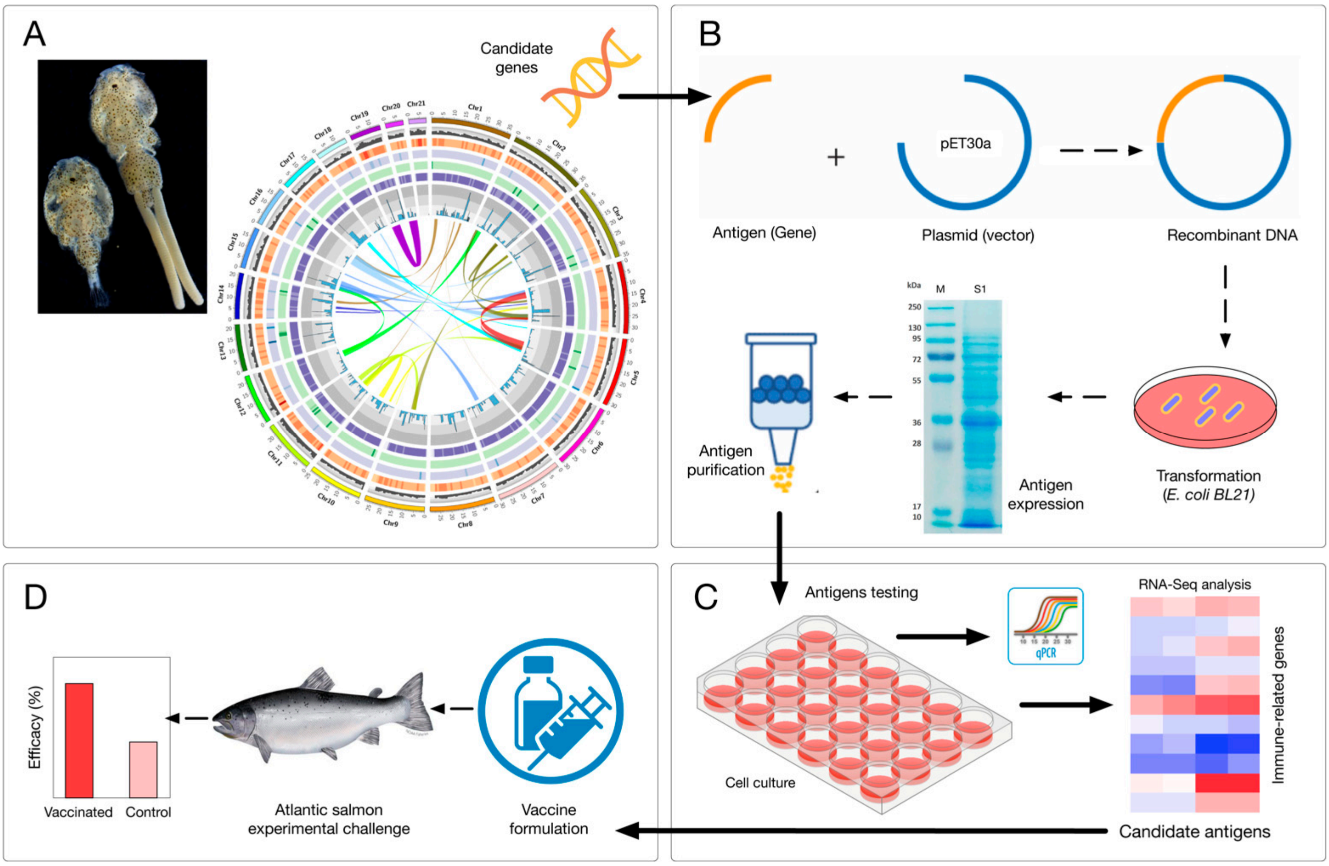

Figure S4. Cell-based antigens prospection workflow for selection of candidate vaccines in Atlantic salmon against *Caligus rogercresseyi*. A) The genome of *C. rogercresseyi* (ASM1338718v1) was used to identify putative candidate vaccine antigen. B) Cloning, expression, and purification of *C. rogercresseyi* Cathepsin protein. C) SHK-1 cell line stimulation with recombinant Cr-Cathepsin protein and transcriptome profiling. D) Experimental fish trial to test the efficacy levels of the candidate vaccine in Atlantic salmon against sea lice.
